# A Retrospective Study Investigating the Incidence and Predisposing Factors of Hospital-Acquired Anemia

**DOI:** 10.1155/2014/634582

**Published:** 2014-12-21

**Authors:** Peter C. Kurniali, Stephanie Curry, Keith W. Brennan, Kim Velletri, Mohammed Shaik, Kenneth A. Schwartz, Elise McCormack

**Affiliations:** ^1^Department of Medicine, Roger Williams Medical Center, Providence, RI, USA; ^2^Department of Medicine, Boston University School of Medicine, Boston, MA, USA; ^3^Division of Hematology Oncology, Michigan State University and Breslin Cancer Center, 401 W. Greenlawn Avenue, Lansing, MI 48910, USA

## Abstract

Hospitalized patients frequently have considerable volumes of blood removed for diagnostic testing which could lead to the development of hospital-acquired anemia. Low hemoglobin levels during hospitalization may result in significant morbidity for patients with underlying cardiorespiratory and other illnesses. We performed a retrospective study and data was collected using a chart review facilitated through an electronic medical record. A total of 479 patients who were not anemic during admission were included in analysis. In our study, we investigated the incidence of HAA and found that, between admission and discharge, 65% of patients dropped their hemoglobin by 1.0 g/dL or more, and 49% of patients developed anemia. We also found that the decrease in hemoglobin between admission and discharge did not differ significantly with smaller phlebotomy tubes. In multivariate analysis, we found that patients with longer hospitalization and those with lower BMI are at higher risk of developing HAA. In conclusion, our study confirms that hospital-acquired anemia is common. More aggressive strategies such as reducing the frequency of blood draws and expanding the use of smaller volume tubes for other laboratory panels may be helpful in reducing the incidence of HAA during hospitalization.

## 1. Introduction

Hospitalized patients frequently have considerable volumes of blood removed for diagnostic testing which may drop their hemoglobin and could lead to the development of hospital-acquired anemia (HAA) [1]. Although the cause of anemia associated with hospitalization is likely multifactorial, iatrogenic anemia due to phlebotomy has been described [1–3]. Low hemoglobin levels during hospitalization may result in significant morbidity for patients with underlying cardiorespiratory and other illnesses [2]. Packed red blood cell transfusion is usually required when the patients become symptomatic or when hemoglobin level is less than 7 g/dL, depending upon comorbid diseases. Blood products transfusion has the risks, including infectious and noninfectious complications [4–6]. Several strategies have been proposed in an attempt to reduce the risk of anemia due to phlebotomy, such as utilizing smaller volume blood tubes for diagnostic testing. However, there is limited research investigating whether a reduction in the size of the phlebotomy tube will reduce the incidence of anemia.

The primary objective of the study was to investigate the incidence of HAA. The impact of reducing the volume of blood taken from hospital patients by using smaller volume phlebotomy tubes on the decrease in hospitalized patients hemoglobin was investigated as a secondary objective. We hypothesized that, following the change in size to a smaller tube, the decrease in hemoglobin during hospitalization will be diminished when compared to a time period before use of the smaller volume tube was initiated.

## 2. Methods

### 2.1. Data Sources

A retrospective study on patients admitted to Roger Williams Medical Center (RWMC) in Providence, RI, was performed on hospital admissions between January 2011 and October 2011.

### 2.2. Reduction of Phlebotomy Tubes

On May 16, 2011, a new smaller phlebotomy tube was implemented at RWMC. The hospital changed the size of the tube used for collecting a basic metabolic panel (BMP) from an 8.5 mL tube (BD 367988) to a 4.5 mL tube (BD 367962), a reduction in volume of 4 mL. This permitted delineation of two separate study groups: those hospitalized before (group A) and those hospitalized after (group B) implementation of reduced volume blood collection tubes. Comparing these two groups allowed us to assess whether the volume of the phlebotomy tube affects the drop in hemoglobin for hospitalized patients.

### 2.3. Eligibility Criteria

We reviewed charts from patients admitted to the general internal medical floor at RWMC between January 2011 and October 2011. Patients over age of 18 admitted to internal medicine service during this period were included in the study. In order to determine whether the decrease in hemoglobin was related to the hospitalization or to their underlying illness, certain patients who met the following criteria were excluded from the study. Those patients who had acute medical conditions that may cause or contribute to the decrease in hemoglobin (including gastrointestinal bleeding, transfusion dependent anemia, a previous history of anemia, hemolysis, hemorrhagic stroke, retroperitoneal bleed, chronic kidney disease/dialysis, or any hematologic malignancy) or were treated with medications that may affect hemoglobin levels (including iron or erythropoietin or chemotherapy) were excluded. Those who had central or peripherally inserted central line placement, who received blood transfusion during hospitalization, and who were hospitalized for less than 2 days or were triaged to surgical, intensive care unit (ICU), cardiac care unit (CCU), and step-down unit (a transition unit for patients who were treated in the ICU or CCU) were also excluded. We also exclude patients who had hemoglobin in the anemic range (less than 13 g/dL for males and less than 12 g/dL for females according to the WHO criteria) on hospital admission.

### 2.4. Statistical Analysis

Data was collected using a chart review facilitated through an electronic medical record. Statistical analysis was done using chi-square test for categorical variable and *t*-test for numerical variable. Patients characteristics were compared based on age group (<65 years and ≥65 years) (Table 1).

In the first part of analysis, the incidence of anemia in hospitalized patients was analyzed using chi-square test. In the second part of analysis, the factors which can predict drop in hemoglobin were assessed using multiple regression analysis using drop of hemoglobin (continuous variable) as a dependent variable and age, race, body mass index (BMI), length of stay, BUN/creatinine, tube size (groups A and B), and comorbidities (hypertension, diabetes mellitus, coronary artery disease, cerebrovascular disease, chronic kidney disease, dyslipidemia, peripheral arterial disease) as independent variables. We used backward selection technique, and the best-fit model was selected after adjusting all the confounding variables. The level of significance was set at *P* < 0.05 for variable in final model. Data were analyzed using the SAS 9.3 (the SAS Corporation, Inc., Cary, NC). Graphics were developed using Prism 6.04 (GraphPad Software, Inc., La Jolla, CA).

## 3. Results

### 3.1. Characteristics of the Patients

**Figure 1:**
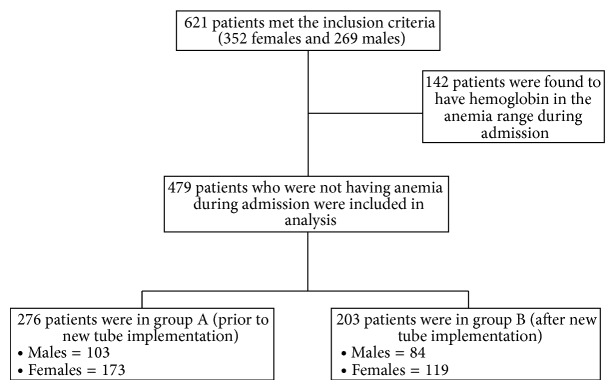
Patients enrollments and selections—detailed.

From January 2011 to October 2011, a total of 4206 hospitalizations to general medical floor were reviewed and 621 hospitalizations met the inclusion criteria. A total of 142 patients out of 621 patients were found to have hemoglobin in the anemic range on hospital admission and were excluded from the analysis. A total of 479 patients who were not anemic during admission were included in the analysis. The effect of a smaller volume phlebotomy tube on the incidence of HAA was evaluated in two groups (276/479 in group A and 203/479 in group B) (Figure 1).

Patients were characterized based on their sex, demographic, comorbidities, length of hospitalization, and kidney function. Patients that were elderly (defined as age ≥ 65) had more comorbidities, longer hospitalizations, and a higher incidence of dehydration on admission (Table 1).

### 3.2. The Drop in Hemoglobin after Hospitalization

After exclusion of patients who were anemic on admission, there were 479 patients, 39 percent men (186/479) and 61 percent women (293/479). Between admission and discharge, 65% of patients (310/479 patients) dropped their hemoglobin by 1.0 g/dL or more, 63% of the men (118/186 patients) and 66% of the women (192/293 patients).

**Figure 2:**
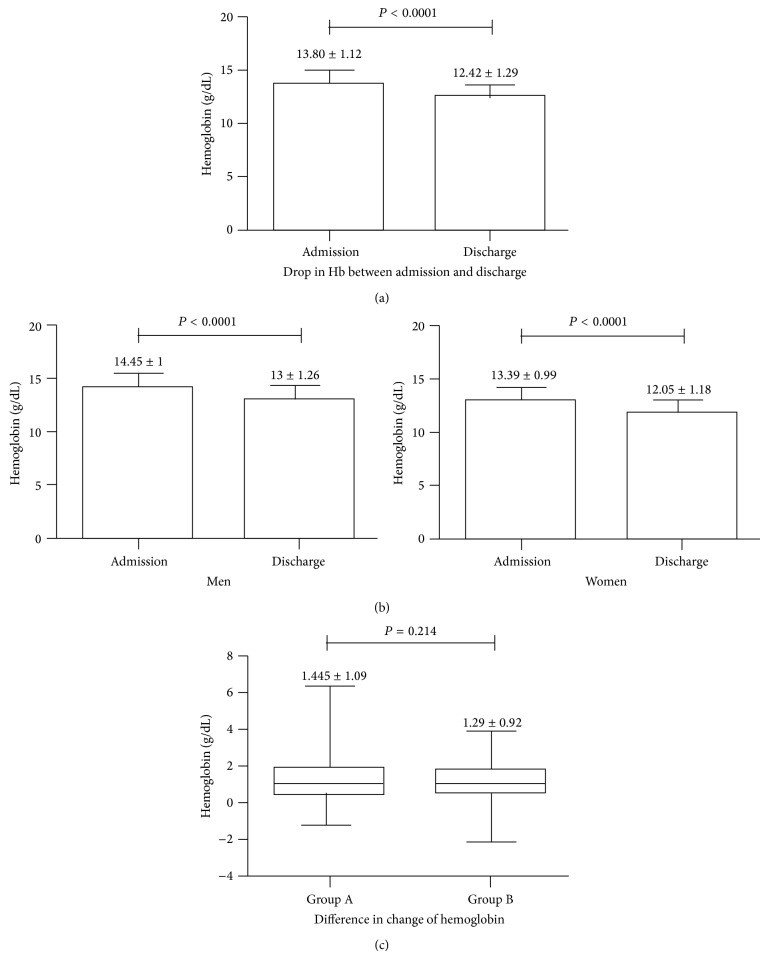
Difference in change and reduction of hemoglobin before and after smaller tube implementation. Hemoglobin dropped significantly between admission and discharge (Figure 2(a)). The drop in hemoglobin between admission and discharge by sex categorization. Both men and women dropped their hemoglobin significantly (Figure 2(b)). Implementation of smaller tube did not significantly reduce the change in hemoglobin (Figure 2(c)).

Upon discharge, 49% of patients (234/479) developed anemia. On average, patients dropped their hemoglobin from 13.8 (on admission) to 12.4 gm/dL (on discharge) (Figure 2(a)). Among this group, 37% (86/234) were men and 63% (148/234 patients) were women. The hemoglobin in men dropped from 14.45 to 13 gm/dL (*P* < 0.0001), while, in women, it dropped from 13.39 to 12.05 gm/dL (*P* < 0.0001) (Figure 2(b)). However, the difference in the rate of anemia by gender was not significant (*P* = 0.36). On average, men and women dropped their hemoglobin by 1.4 and 1.3 g/dL, respectively.

An age-group analysis showed that 54% (138 out of 255) of patients aged ≥ 65 years became anemic during hospitalization compared to 42% (96 out of 224) of patients aged < 65 years (*P* = 0.01).

### 3.3. Difference in Change of Hemoglobin before and after Decreased Volume Tube Implementation

Eleven percent of blood draws in this hospital included BMP, in which the volume of phlebotomy tube was reduced from 8 cc to 4 cc. The decrease in hemoglobin between admission and discharge did not differ significantly between group A and group B (1.44 ± 1.09 versus 1.29 ± 0.92, *P* = 0.214) (Figure 2(c)).

### 3.4. Multivariate Analysis of the Drop in Hemoglobin

We performed multiple linear regression analysis on patients to evaluate factors contributing to the drop in hemoglobin. Even though elderly patients had a higher incidence of HAA compared to the younger counterparts, after adjusting with other factors, such as comorbidities and body mass index, the difference was not statistically significant. After adjusting the age, sex, length of hospitalization, BUN/creatinine ratio on admission (to evaluate the effect of dehydration), comorbidities, and BMI, we found that the two main factors which can predict the drop in hemoglobin in hospitalized patients were the length of stay and BMI. One-day increase in length of hospital stay decreases the hemoglobin by 0.06 (±0.03) mg/dL under the assumption that the other variables are held constant. Using the same statistical constraints, an increase in BMI by 10 would decrease the drop of hemoglobin by 0.1 mg/dL (Table 2).

## 4. Discussion

There have been few studies evaluating incidence of anemia during admission to a general medical floor. Our study is the first to demonstrate that decreasing the volume of blood used for phlebotomy failed to decrease the incidence and severity of HAA. Hospitalization is associated with a significant decrease in hemoglobin. In our study, about 65 percent of patients dropped their hemoglobin by at least 1 gm/dL or more, and 45 percent of patients became anemic during hospitalization. Patients who had longer hospitalization and those who had lower BMI had a greater decrease in hemoglobin.

Patients admitted to the hospital may also have dehydration due to their medical conditions. These patients often received intravenous hydration, which may cause hemodilution leading to a reduction in hemoglobin [7, 8]. In our study, BUN/creatinine ratio, which is considered a surrogate indicator for hydration status, did not have significant effect on drop of hemoglobin.

The mechanism of hospital-acquired anemia may depend upon the amount of blood draws for diagnostic studies as well as decreased red cell production secondary to the patients associated comorbidities. The patients primary illness could include inflammation or infection with a decreased RBC production commonly referred to as the anemia of chronic disease. The high frequency of anemia developing during hospitalization suggests that both decreased production and high volume of blood withdrawn for diagnostic purposes contribute to the anemia.

The results of our study were similar to previous studies showing that length of stay is an important predictor for the development of HAA [9]. The reported incidence of HAA from previous studies varies from 25% to 74%, depending on the study population (i.e., acute coronary syndrome and kidney disease) [3, 9–11].

Our findings supported the theory that the amount of blood drawn correlates with the development of HAA. The longer the patients were hospitalized, the more likely they would have increased amounts of blood draws. Using smaller volume tubes for BMP, which constituted only 11% of blood draws in this institution, did not significantly affect the drop of hemoglobin. However, the effect might have been significant if the smaller tube was applied to a larger proportion of blood drawn (i.e., complete blood count or cardiac enzymes).

Potential limitations of this study are that it was retrospective and did not include serial measurements of reticulocytes as an index of red cell production. We also did not measure the exact amount of blood lost during phlebotomy as well as the frequency of phlebotomy and timing in each patient. This study was performed in single institution and may not be applicable to other clinical centers. We are aware of variability for the development of HAA among medical institutions. A recent study showed that teaching institutions had a lower risk of HAA [12].

In conclusion, our study confirms that hospital-acquired anemia is common. Almost half of our studied hospitalized patients developed HAA. Patients with longer hospitalization and those with lower BMI are at higher risk of developing HAA. More aggressive strategies, such as reducing the frequency of blood draws and expanding the use of smaller volume tubes for other laboratory panels, may be helpful in reducing the incidence of HAA during hospitalization. It is important for physicians to be aware of HAA and to reduce unnecessary blood draws that may contribute to the high incidence of HAA.

## Figures and Tables

**Table 1:** Baseline characteristics of the patients.

	<65	≥65	*P* value
Patients who meet the inclusion criteria	224	255	
Sex			0.002
M	103 (46%)	83 (33%)
F	121 (54%)	172 (67%)
Race			0.001
Caucasian	167	231
AA	21	7
Hispanic	25	13
Other	11	4
Length of stay	2.92 ± 1.3	3.25 ± 1.6	0.01
Comorbidities			0.001
0	69	16
1	57	45
2	44	81
3	33	55
≥4	21	58
Admission BUN	14.9 ± 6.5	22.07 ± 10	0.001
Admission creatinine	0.85 ± 0.2	0.94 ± 0.3	0.0009
BUN/creatinine ratio	18.05 ± 8.6	23.7 ± 8.17	0.0001

**Table 2:** Multilinear regression analysis: factors influencing the drop of hemoglobin in hospitalized patients.

Independent variable	**β**-estimate (±SE)	Number of patients used in analysis	*P* value
Age ≥65 y versus <65 y	0.166 (±0.097)	479	0.08
Male versus female	−0.058 (±0.098)	479	0.55
BMI	−0.014 (±0.005)	479	0.005^*^
Length of stay	0.067 (±0.030)	479	0.02^*^
BUN/CR	−0.005 (±0.005)	479	0.35
Group A versus B	−0.162 (±0.094)	479	0.08
Comorbidities (<3 versus ≥3)	−0.020 (±0.100)	479	0.77
White versus others	0.484 (±0.276)	479	0.08
African American versus others	0.541 (±0.330)	479	0.10
Hispanic versus others	0.490 (±0.316)	479	0.12

^*^Statistically significant.

## References

[1] Thavendiranathan P., Bagai A., Ebidia A., Detsky A. S., Choudhry N. K. (2005). Do blood tests cause anemia in hospitalized patients? The effect of diagnostic phlebotomy on hemoglobin and hematocrit levels. *Journal of General Internal Medicine*.

[2] Pabla L., Watkins E., Doughty H. A. (2009). A study of blood loss from phlebotomy in renal medical inpatients. *Transfusion Medicine*.

[3] Salisbury A. C., Reid K. J., Alexander K. P., Masoudi F. A., Lai S.-M., Chan P. S., Bach R. G., Wang T. Y., Spertus J. A., Kosiborod M. (2011). Diagnostic blood loss from phlebotomy and hospital-acquired anemia during acute myocardial infarction. *Archives of Internal Medicine*.

[4] Hébert P. C. (1999). Anemia and red cell transfusion in critical care. Transfusion Requirements in Critical Care Investigators and the Canadian Critical Care Trials Group. *Minerva Anestesiologica*.

[5] Hébert P. C., Wells G., Blajchman M. A., Marshall J., Martin C., Pagliarello G., Tweeddale M., Schweitzer I., Yetisir E. (1999). A multicenter, randomized, controlled clinical trial of transfusion requirements in critical care. Transfusion Requirements in Critical Care Investigators, Canadian Critical Care Trials Group. *The New England Journal of Medicine*.

[6] Rao S. V., Jollis J. G., Harrington R. A., Granger C. B., Newby L. K., Armstrong P. W., Moliterno D. J., Lindblad L., Pieper K., Topol E. J., Stamler J. S., Califf R. M. (2004). Relationship of blood transfusion and clinical outcomes in patients with acute coronary syndromes. *Journal of the American Medical Association*.

[7] Rasouli M., Kiasari A. M., Arab S. (2008). Indicators of dehydration and haemoconcentration are associated with the prevalence and severity of coronary artery disease. *Clinical and Experimental Pharmacology and Physiology*.

[8] Shirreffs S. M. (2000). Markers of hydration status. *The Journal of Sports Medicine and Physical Fitness*.

[9] Koch C. G., Li L., Sun Z., Hixson E. D., Tang A., Phillips S. C., Blackstone E. H., Henderson J. M. (2013). Hospital-acquired anemia: prevalence, outcomes, and healthcare implications. *Journal of Hospital Medicine*.

[10] Meroño O., Cladellas M., Recasens L., Garcia-Garcia C., Ribas N., Bazan V., Farré N., Sainz Á., Comin J., Bruguera J. (2012). In-hospital acquired anemia in acute coronary syndrome. Predictors, in-hospital prognosis and one-year mortality. *Revista Espanola de Cardiologia*.

[11] Choi J. S., Kim Y. A., Kang Y. U., Kim C. S., Bae E. H., Ma S. K., Ahn Y.-K., Jeong M. H., Kim S. W. (2013). Clinical impact of hospital-acquired anemia in association with acute kidney injury and chronic kidney disease in patients with acute myocardial infarction. *PLoS ONE*.

[12] Salisbury A. C., Reid K. J., Amin A. P., Spertus J. A., Kosiborod M. (2014). Variation in the incidence of hospital-acquired anemia during hospitalization with acute myocardial infarction (Data from 57 US Hospitals). *American Journal of Cardiology*.

